# 577 Impact of Heparin and Enoxaparin Anticoagulant Prophylaxis on Improving Acute Mortality in Burn Patients

**DOI:** 10.1093/jbcr/iraf019.206

**Published:** 2025-04-01

**Authors:** Manav Patel, Shawn Lim, Victoria Cuello, Joshua Lewis, Michael Erickson, Steven Wolf, Juquan Song

**Affiliations:** University of Texas Medical Branch, John Sealy School of Medicine; University of Texas Medical Branch; University of Texas Medical Branch; University of Texas Medical Branch; Shriners Children’s - Galveston; University of Texas Medical Branch; University of Texas Medical Branch

## Abstract

**Introduction:**

Burn patients have a higher chance of developing thromboembolic complications leading to worsened mortality rates so prophylactic anticoagulation is important. Anticoagulants such as enoxaparin, a low molecular weight heparin (LMWH), and unfractionated heparin (UFH) have been frequently used as chemical prophylactic treatments of thromboembolisms. Enoxaparin has been shown to have lower mortality and higher efficacy in surgical patients and coronary artery disease patients. The aim of the study is to assess mortality and compare the safety of enoxaparin and heparin in acute burn patients.

**Methods:**

A retrospective cohort study of 26227 burn patients was conducted using the TriNetX database. Patients were divided into two cohorts: those receiving only unfractionated heparin (Cohort 1) and those receiving only enoxaparin (Cohort 2) prophylaxis within 24 hours after burn injury. Cohorts were matched with 1:1 propensity score matching to correct for differences in age, gender, ethnicity, race, burn severity, diabetes mellitus, acute myocardial infarction, stroke, and central line venous catheter placement. Outcomes assessed included mortality and deep vein thrombosis (DVT) within a month (30 days).

**Results:**

Post-matching, there were 7790 patients in each cohort. The heparin cohort demonstrated a significantly higher 30-day mortality rate compared to the enoxaparin cohort (RR = 2.321, 95% CI [1.878, 2.869], p < 0.05). The relative risk ratio for DVT in heparin cohort was slightly higher but not significant (RR = 1.303, 95% CI [0.572, 2.971], p=0.527).

**Conclusions:**

Prophylactic anticoagulation with enoxaparin is associated with a significant lower 30-day mortality risk compared to unfractionated heparin.

**Applicability of Research to Practice:**

The findings of this study provide important clinical insights that can directly influence the management of thromboembolic prophylaxis in burn patients. Given that enoxaparin is associated with significantly lower 30-day mortality compared to unfractionated heparin, burn centers and critical care units should consider incorporating enoxaparin as the preferred anticoagulant in their prophylaxis protocols.

**Funding for the Study:**

N/A

